# Ligands specify estrogen receptor alpha nuclear localization and degradation

**DOI:** 10.1186/1471-2121-11-98

**Published:** 2010-12-10

**Authors:** Silvia Kocanova, Mahta Mazaheri, Stéphanie Caze-Subra, Kerstin Bystricky

**Affiliations:** 1 Université de Toulouse; UPS; Laboratoire de Biologie Moléculaire Eucaryote; F-31062 Toulouse, France; 2 CNRS; LBME; F-31000 Toulouse, France

## Abstract

**Background:**

The estrogen receptor alpha (ERα) is found predominately in the nucleus, both in hormone stimulated and untreated cells. Intracellular distribution of the ERα changes in the presence of agonists but the impact of different antiestrogens on the fate of ERα is a matter of debate.

**Results:**

A MCF-7 cell line stably expressing GFP-tagged human ERα (SK19 cell line) was created to examine the localization of ligand-bound GFP-ERα. We combined digitonin-based cell fractionation analyses with fluorescence and immuno-electron microscopy to determine the intracellular distribution of ligand-bound ERα and/or GFP-ERα.

Using fluorescence- and electron microscopy we demonstrate that both endogenous ERα and GFP-ERα form numerous nuclear focal accumulations upon addition of agonist, 17β-estradiol (E2), and pure antagonists (selective estrogen regulator disruptor; SERD), ICI 182,780 or RU58,668, while in the presence of partial antagonists (selective estrogen regulator modulator; SERM), 4-hydroxytamoxifen (OHT) or RU39,411, diffuse nuclear staining persisted.

Digitonin based cell fractionation analyses confirmed that endogenous ERα and GFP-ERα predominantly reside in the nuclear fraction. Overall ERα protein levels were reduced after estradiol treatment. In the presence of SERMs ERα was stabilized in the nuclear soluble fraction, while in the presence of SERDs protein levels decreased drastically and the remaining ERα was largely found in a nuclear insoluble fraction. mRNA levels of *ESR1 *were reduced compared to untreated cells in the presence of all ligands tested, including E2. E2 and SERDs induced ERα degradation occurred in distinct nuclear foci composed of ERα and the proteasome providing a simple explanation for ERα sequestration in the nucleus.

**Conclusions:**

Our results indicate that chemical structure of ligands directly affect the nuclear fate and protein turnover of the estrogen receptor alpha independently of their impact on transcription. These findings provide a molecular basis for the selection of antiestrogen compounds issue from pharmacological studies aimed at improving treatment of breast cancer.

## Background

The estrogen receptor alpha (ERα) is a member of the steroid nuclear receptor family. The gene coding for ERα (*ESR1*) is regulated by seven different promoters that yield different transcripts, making it one of the most complex genes in the human genome [1]. Several splice variants have been described for estrogen receptor α, but whether all these variants are expressed as functional proteins with biological functions is not clear [2,3]. In the classic pathway ERα undergoes a conformational change in the presence of estradiol, which leads to association with ERα target genes via direct binding to regulatory elements and modulation of their expression. This basic mechanism is influenced by other regulatory factors including alternate receptor isoforms, and the stoichiometry of coactivator and corepressor proteins. Coactivators have a common LXXLL motif [4] and after binding to the AF-2 domain of ERα, facilitate recruitment of other factors [5]. Mutation analysis combined with crystallographic studies demonstrated that receptor-coactivator interactions are mediated through the ERα helix12 and the LXXLL motif of coactivators [6]. 4-hydroxytamoxifen (OHT) acts by blocking AF-2 activity so it is an antagonist in cells where AF-2 is dominant and a partial agonist where AF-1 is dominant [7]. Fulvestrant/ICI 182,780 (ICI) is known to block both, AF-2 and AF-1 activities.

Estrogens have a proliferative effect on various tissues, including the breast. Thus ERα plays a key role in mammary tumour development. In mammary cells, the effects of 17β-estradiol (E2) can be antagonized by compounds such as OHT, a tamoxifen metabolite that is a selective estrogen receptor modulator (SERM), and ICI, a selective estrogen receptor disruptor (SERD). OHT has partial agonist activity, depending on the tissue and response examined while ICI compounds are totally devoid of agonist activity in the models studied to date [8-10]. ERα-OHT complexes accumulate in nuclei and ICI treatment provokes rapid degradation of the ERα-ICI complex by the nuclear proteasome [11,12].

Intracellular levels of ERα are downregulated in the presence of E2, its cognate ligand, through the ubiquitin/proteasome (Ub/26S) pathway [10]. Polyubiquitination of liganded ERα is catalyzed by at least three enzymes: the ubiquitine-activating enzyme E1 activated ubiquitin is conjugated by E2 with lysine residues through an isopeptide bond by the E3 ubiquitin ligase. Polyubiquitinated ERα is then directed to the proteasome for degradation [13,14]. Most known ubiquitin attachment sites reside within the C-terminus of the ERα. Berry et al. recently also identified two receptor lysines, K302 and K303 in the hinge region of ERα which are involved in E2 mediated and ICI induced ERα degradation in breast cancer cells [15]. Although ER-dependent transcription regulation and proteasome-mediated degradation of the ERα are linked [16], transcription per se is not required for ERα degradation and assembly of the transcription-initiation complex is sufficient to target ERα for degradation by the nuclear fraction of the proteasome [13]. Using immunocytochemical studies it was shown that ERα resides predominantly in the nucleus both in presence or absence of hormone [17]. Maruvada et al. [18] determined that a small proportion of transiently transfected GFP- ERα exists in the cytoplasm in the absence of hormone. They proposed that unbound ERα shuttles between the cytoplasm and nucleus in living cells. Estradiol and E2 antagonists affect ERα protein turnover rates and modulate transcription of ERα target genes [19,20]. It has been shown that E2 induced degradation of ERα is necessary for its ability to rapidly activate transcription [21]. Interestingly, two chemically different SERDs (ICI and RU58,668) competitively inhibit estradiol-mediated activation by ERα and induce rapid down-regulation of the receptor [22,23]. In contrast, in the presence of tamoxifen ERα protein levels increase, although the effect of OHT on transcription is similar to the one observed for SERD's in MCF-7 cells [19].

In the present study we determine the impact of different ligands on nucleocytoplasmic shuttling of ERα and examine the relationship between localization and proteolysis, two mechanisms involved in ERα-mediated regulation in MCF-7 cells. To achieve this goal, we determined ERα protein concentration, subnuclear localization of ERα with relationship to the proteasome, and the level of *ESR1 *transcription upon treatment with various antiestrogens.

## Results

### Ligands regulate ERα protein levels and transcription rates independently

**Figure 1:**
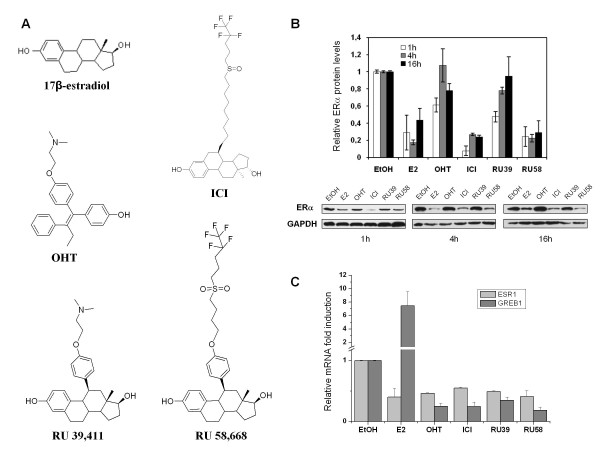
**Protein and mRNA levels of ligand bound ERα in MCF-7 cells**. A) Chemical structure of 17β-estradiol, SERMs (OHT, RU39) and SERDs (ICI, RU58). B) Western blot and quantification of ERα protein levels after 1 h, 4 h and 16 h treatment with 10 nM E2, 1 μM ICI, 1 μM RU58, 1 μM OHT and 1 μM RU39 relative to ERα protein levels in untreated (EtOH) MCF-7 cells. 2 μg of total protein were loaded. Relative protein levels from three independent experiments were quantified. C) Total RNA was extracted from MCF-7 cells treated or not for 16 h with 10 nM E2 or anti-estrogens. Relative expression level of the *ESR1 *and *GREB1 *genes was analyzed by qRT-PCR. *TBP *served as an internal control (see Methods). Data shown are an average of three independent experiments, error bars represent ± S.E. mean.

We first examined the kinetics of ERα protein turnover in MCF-7 cells following treatment with estradiol (E2), two SERMs (4-hydroxytamoxifen or OHT and RU39,411 or RU39) and two SERDs (ICI 182,780 or ICI and RU58,668 or RU58). It has been proposed that ligand dependent ERα regulation may result from the presence a long aliphatic side chain on steroid core. Thus in this study we selected RU39 and RU58 which are derivatives of 17β-estradiol but with different side chains. RU39 has a dimethyl-amino-ethoxy-phenyl side chain similar to the one in tamoxifen, while RU58 has a bulky hydrophobic side chain similar to the one in Fulvestrant (ICI) (Figure 1A). ERα protein levels in E2, ICI and RU58 treated MCF-7 cells rapidly decreased (Figure 1B). Time course experiments showed that 1 h after E2 induction, the detected amount of ERα protein accounted for only 40% of ERα levels before treatment; after 4 h, ERα levels were as low as 20% of the quantity of ERα present in untreated cells, and after 16 h ERα protein remained at a level equivalent to the one observed 1 h after addition of E2. Treatment with SERDs (ICI and RU58) resulted in >70% reduction of ERα protein levels after 1 h, 4 h and even 16 h reaching 95% after 1 h exposure to ICI (Figure 1B). Treatment of MCF-7 cells with OHT or RU39 (Figure 1B), two compounds classified as SERM, reduced from 40% to 50% of ERα protein levels at the initial 1 h time-point and about 20% after 16 h and 4 h treatment with OHT and RU39, respectively. In addition, ERα protein levels were almost equivalent to the ones detected in untreated cells after 4 h or 16 h culture in the presence of OHT or RU39. Hence, ERα protein levels are stabilized by SERMs.

To assess whether changes in protein levels reflect variations of ERα protein stability or of transcription rates of the *ESR1 *gene, we quantified ERα mRNA accumulated following 16 h treatment with the different compounds (Figure 1C). *ESR1 *mRNA expression was greatly reduced after treatment with ERα ligands. In the presence of E2, only 40% of *ESR1 *mRNA could be recovered. Similarly, treatment with SERMs and SERDs repressed *ESR1 *mRNA transcription by 45%-60% relative to untreated MCF-7 cells. Despite the fact that a reduction in ERα protein levels was readily detectable after 1 h and significant after 16 h (Figure 1B), we observed that E2 induced a 7 to 10 fold increase in mRNA levels of the ERα-target gene *GREB1 *compared to mock treated cells (Figure 1C). *GREB1 *transcription was inhibited by SERMs and SERDs (> 40% reduction compared to untreated cells; Figure 1C). These results were expected since E2 is known to activate this ERα target gene, while SERMs and SERDs are antiestrogens and thus repress *GREB1 *transcription in ERα positive mammary tumour cells [20].

Thus, in MCF-7 cells, variations in ERα protein levels do not necessarily correlate with *ESR1 *transcription in the presence of ligands. We note that the decrease in ERα protein levels is more pronounced after treatment with SERDs than after addition of E2, while the effect of hormone and SERDs on *ESR1 *mRNA accumulation was comparable. These results indicate that reduction of ERα protein levels following treatment with SERDs cannot be solely attributed to decreased *ESR1 *mRNA levels. SERDs apparently act both on transcription of the *ESR1 *gene and on ERα protein turnover. In contrast, ERα protein levels appear stable after 16 h treatment with SERMs (data not shown) despite reduced *ESR1 *expression levels. This suggests that binding to SERMs stabilizes the ERα.

### Ligands directly affect intracellular distribution and stability of ERα

**Figure 2:**
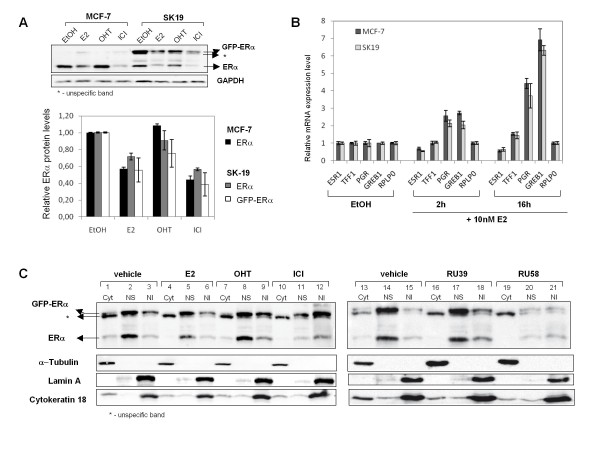
**Nuclear accumulation and degradation of the estrogen receptor alpha in MCF7 and SK19 cells**. A) Protein levels of endogenous ERα and GFP-ERα in MCF-7 and SK19 cells in response to E2 and to anti-estrogens. The SK19 cell line was generated from MCF-7 cells by stably transfecting a GFP-ERα expression vector (pEGFP-C2-hERα). B) Total RNA was extracted from SK19 and MCF-7 cells treated or not for 2 h and 16 h with 10 nM E2. Relative expression level of the *ESR1*, *TFF1, GREB1 *and *PGR *genes in SK19 cells was analyzed by qRT-PCR and compared to gene-expression regulation in MCF-7 cells. *RPLP0 *served as an internal control (see Methods). Data shown are an average of two independent experiments, error bars represent ± S.E. mean. C) Cellular distribution of ERα and GFP-ERα from digitonin based cellular fractionation experiments. SK19 cells were treated with drugs for 3 h. Nuclear fractions of untreated and treated cells with 10 nM E2, 1 μM OHT, 1 μM ICI, 1 μM RU39 and 1 μM RU58, were isolated as described in "Methods". Nuclear content of ERα and GFP-ERα was analyzed by Western Blotting using anti-ERα antibodies. The specific subcellular proteins, α-tubulin for cytoplasmic fraction (Cyt), Lamin A for nuclear insoluble fraction (NI) and cytokeratin 18 for nuclear soluble fraction (NS) are loading controls for the different cellular compartments. Results shown are representative of at least 3 independent experiments.

SERMs and SERDs can be distinguished based on molecular mechanisms [9]. To unambiguously determine localization of the estrogen receptor and its intracellular trafficking in response to treatment with various ligands we established a MCF-7 cell line stably expressing GFP-ERα from a CMV promoter. It was previously shown that transiently expressed GFP-ERα is functional using an estrogen response element driven luciferase reporter gene [24]. Expression of GFP-ERα in MCF-7 cells did reportedly not alter cell cycle progression and GFP-ERα participated in estrogen target gene regulation similarly to endogenous ERα [25]. We tagged the N-terminus of the human ERα with the S65T variant of GFP for transfection and stable integration in MCF-7 cells. Several clones were recovered and screened for total GFP-ERα protein content after treatment with E2, OHT or ICI using fluorescence microscopy and western blots. Here, we selected a MCF-7 derived clone (SK19) expressing GFP-ERα in which changes in endogenous ERα protein levels in response to a 4 h treatment with E2, OHT and ICI were identical to the ones observed in MCF-7 cells (compare lanes labeled ERα in MCF-7 and SK19 cells in Figure 2A). In addition, mRNA expression levels of some ERα target genes, *ESR1, TFF1/pS2, GREB1 *and *PGR*, were verified in the selected clone SK19 and compared to gene expression levels in MCF-7 cells (Figure 2B). mRNA levels of the progesterone receptor gene (*PGR*) and *GREB1 *increased rapidly after addition of 10 nM E2 to cells grown in steroid free medium to reach 2.2 to 2.8 fold (after 2 h) for both genes, and after 16 h to reach from 3.8 to 4.6 fold for *PGR *and 6.3 to 7.0 fold for *GREB1 *gene, in MCF-7 and SK19 cells respectively. *TFF1 *mRNA also accumulated after 16 h E2 treatment to reach 1.5 fold in both cell lines. As expected, *ESR1 *transcription was reduced in the presence of E2. The *RPLPO *gene is not a target of ERα and its expression levels were insensitive to hormone addition. Expression levels of all tested genes were similar in SK19 and MCF-7 cells. Thus the presence of GFP-ERα does not alter hormone-responsiveness at the transcriptional level.

In SK19 cells, GFP-ERα protein accounted for 50% of total ERα (GFP-ERα and endogenous ERα) in untreated cells. In the presence of E2 both GFP-ERα and endogenous ERα protein levels are reduced (Figure 2A). The CMV promoter being insensitive to E2 and antiestrogens, GFP-ERα protein levels are unlikely to be transcriptionally regulated. This observation together with the results shown in Figure 1 provides evidence that ERα protein turnover is regulated directly by binding of the receptor to ligands and its subsequent degradation.

Previous studies have demonstrated that GFP-ERα resides predominantly in the nucleus in transiently transfected mammary tumour cell lines [24], Hela cells [26,27] and in MCF-7 cells expressing GFP-ERα from an inducible promoter [25]. These microscopy based observations largely contradict results based on cellular fractionation which suggest that large amounts of ERα, in the absence or the presence of ligands, associate with the cytoplasmic fraction [19]. It has been proposed that the relative amount of cytoplasmic ERα is indicative of the mechanism of action of certain antiestrogens [19]. Commonly used cell fractionation protocols include a detergent based extraction step. Importantly, ERα and other nuclear receptors such as the glucocorticoid receptor (GR) are easily extracted from the nucleus in the presence of low concentrations of detergents such as NP40 (V. Marsaud and H. Richard-Foy, unpublished observations). As a consequence, apparent enrichment of ERα or GR in the cytoplasm likely results from the extraction protocol rather than a specific behavior of nuclear receptors. Here, we used a digitonin based cell fractionation protocol to determine the distribution of unbound and ligand-bound ERα and GFP-ERα in different cellular compartments (Figure 2C). Effectiveness of the fractionation protocol (for details see Methods) was confirmed using lamin A for the nuclear insoluble fractions, cytokeratin 18 for the nuclear and cytoplasmic fractions, and α-tubulin for the cytoplasmic fraction (Figure 2C). Treatment of cells with E2 and various antiestrogens did not affect cellular distribution of these proteins. We found that endogenous ERα associates predominantly with the nuclear fraction in the SK19 cells. In untreated cells, the part of ERα retained in the cytoplasm corresponded to ~20% of total endogenous ERα detected using the HC-20 antibody (Figure 2C, *lanes 1 and 13*). Similarly, the bulk of GFP-ERα, detected using either the HC-20 antibody or an antibody directed against GFP, was found in the nucleus. Following addition of E2, we note an overall decrease in ERα protein levels that could mainly be attributed to a reduction in nuclear ERα (Figure 2C, *lanes 4-6*). Treatment of SK19 cells with SERDs, ICI or RU58 leads to a decrease in overall ERα protein levels as shown for MCF-7 cells in Figure 1B. Notably, the remaining ERα was concentrated in the nuclear insoluble fraction which corresponded to ≥40% of total ERα in the presence of either ICI or RU58 (Figure 2C, *lanes 12 and 21*) suggesting that the nuclear soluble fraction was rapidly degraded. In contrast, we found that treatment with OHT and RU39 (Figure 2C, *lanes 7-9 and 16-18*) resulted in a cellular distribution similar to the one observed in untreated cells (Figure 2C, *lanes 1-3*) where at least 50% of ERα protein remained in a soluble nuclear compartment. Our cellular fractionation protocol is robust since the effects of various ligands are reproducible inside each category: OHT and RU39 induce the same effect on ERα protein distribution and this effect is distinct from the one of ICI and RU58. In addition, we show that occupation of different cellular compartments by GFP-ERα reflected the localization of endogenous ERα as detected by fluorescence imaging (see below).

### Ligands induce specific intracellular relocalization of GFP-ERα

**Figure 3:**
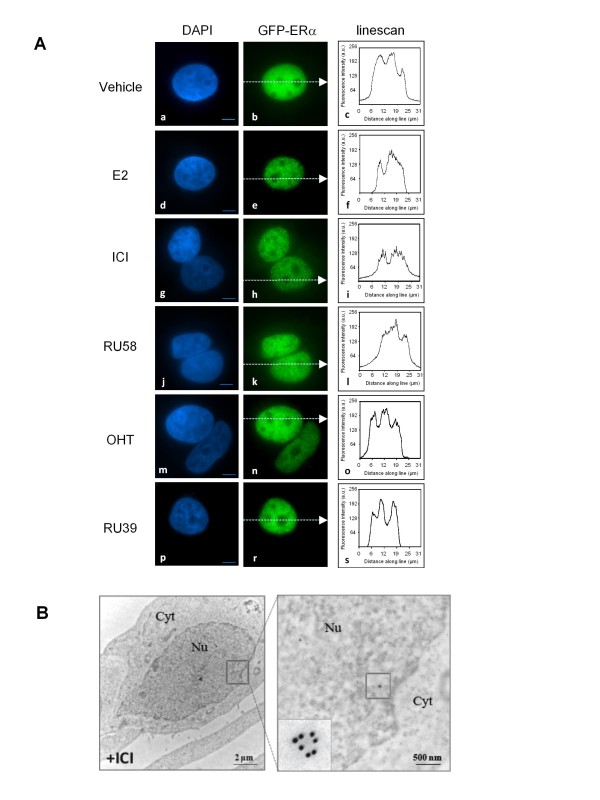
**Estrogen, SERDs and SERMs induce distinct intracellular behavior of ERα**. A) SK19 cells were incubated for 1 h with E2 and different anti-estrogens (SERDs or SERMs), fixed, stained with DAPI and examined by fluorescence microscopy using an Olympus IX-81 inverted microscope. GFP-ERα forms intranuclear foci in the presence of 10 nM E2, 1 μM RU58 and 1 μM ICI, but remains uniformly distributed in the nucleus after addition of 1 μM OHT and 1 μM RU39. Linescans indicate relative fluorescence intensities in the cytoplasm and the nucleus in the cells imaged using identical parameters. Pictures are representative of at least three independent experiments. Bar, 5 μm. B) Representative electron micrograph of MCF-7 cells treated with 1 μM ICI for 1 h. Inset indicates formation of ERα clusters in the nucleus after ICI addition. Bar, 2 μm and 500 nm.

GFP-ERα can be visualized in SK19 cells using conventional wide-field microscopy. SK19 cells were cultured on conventional glass microscopy coverslips in phenol-red free media for 3 days. Culture conditions were identical to conditions used for cell fractionation, immunoblotting or RNA extraction prior to RT-qPCR. Figure 3A shows representative images of SK19 cells treated or not with E2, SERMs and SERDs. We note that in the SK19 cell line GFP-ERα was excluded from the nucleoli, as previously observed for the cellular distribution of endogenous ERα in MCF-7 cells [28,29] and of transiently transfected GFP-ERα [24,26,30], under all conditions tested. Exposure times were identical for all conditions examined by fluorescence microscopy.

In untreated cells, ERα was uniformly distributed in the nucleus (compare GFP-ERα fluorescence (Figure 3Ab), to the DAPI (4',6-diamidino-2-phenylindole) nuclear stain in Figure 3Aa). A linear scan across the entire field including cytoplasm and nucleus (Figure 3Ac) shows that the cytoplasmic GFP-ERα fluorescence was barely above background (~15% of the maximum fluorescence intensity detected in the nucleus) which correlates with observations from cell fractionation experiments (Figure 2C, *lane 1*). In the presence of E2, GFP-ERα rapidly relocalized to accumulate in numerous foci scattered throughout the nucleoplasm (Figure 3Ae). In E2 treated cells, no GFP-ERα fluorescence could be detected in the cytoplasm (see linescan Figure 3Af). In contrast, after 1 h treatment with SERMs, OHT or RU39, we did not observe any intranuclear reorganization of GFP-ERα compared to untreated cells. This observation also correlates with our fractionation experiments (Figure 2C, *lanes 1-3 *compare to *lanes 7-8 *and *16-18*). GFP-ERα staining remained diffuse with fluorescence intensity comparable to mock cells (Figure 3A b, n, r and corresponding linescans Figure 3A c, o, s). However, again no cytoplasmic GFP-ERα could be detected.

The distribution of the intensity of the fluorescent signals was determined within nuclei excluding the nucleolus. The frequency of pixels with respect to their intensity allows to calculate a coefficient of variation (CV). In cells treated with SERMs the CV was comparable to the one in control cells while the CV was 2 to 3 fold higher in cells exposed to E2 or SERDs (Table 1). This quantitive measure strengthens our observation that ERα accumulates in intranuclear foci when bound to E2 or SERDs but not in the presence of SERMs.

**Table 1:** Mean coefficient of variation and its standard deviation in MCF-7 cells treated with E2 and anti-estrogens.

MCF-7 cells	EtOH	E2	ICI	RU58	OHT	RU39
**CV** **(± SD)**	0,0248 (± 0,008)	0,061 (± 0,013)	0,0575 (± 0,019)	0,0622 (± 0,019)	0,0351 (± 0,009)	0,0425 (± 0,013)

Coefficients of variation of fluorescent intensities were measured after treatment with 10 nM E2 and anti-estrogens as described in *Methods*. The distribution of the intensity of the fluorescent signals was determined within nuclei excluding the nucleolus in MCF-7 cells (n = 15 - 20 cells).

Upon exposure to SERDs, both ICI and RU58, GFP-ERα accumulated at numerous sites, reminiscent of the ones observed in the presence of E2 (Figure 3A h, k and 3Ae). We ascertained that the fluorescent foci detected in SK19 cells correspond to an accumulation of endogeneous ERα using immuno-electron microscopy of MCF-7 cells. Several immunogold labeled ERα molecules were frequently detected within ~100 nm distance from each other in 80 nm thin sections of E2 or ICI treated cells (Figure 3B).

In addition, in SK19 cells, the maximum fluorescence intensity measured after E2 and ICI treatments decreased by 20-40% as compared to untreated cells consistent with degradation of GFP-ERα (Figure 3Af and 3Ai compare to 3A*c*). The effects of ICI and RU58 were indistinguishable suggesting that both molecules operate via similar molecular mechanisms despite significant structural differences [19].

### Proteasome-dependent degradation of ERα bound to E2 or SERDs

ERα is a short-lived protein (half-life of >3 h for unbound ERα and ~ 1-3 h for ligand-bound ERα) [10,31]. ERα degradation occurs in presence of natural ligands (E2) or pure antiestrogens such as ICI in a proteasome dependent manner [13,14,32].

**Figure 4:**
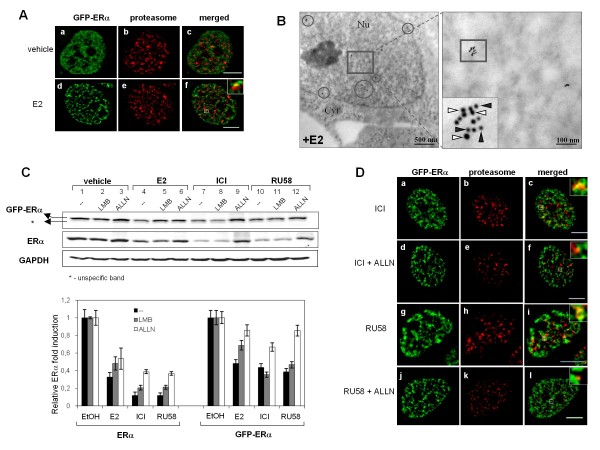
**Nuclear Proteasome-GFP-ERα contacts are frequent in the presence of E2 and SERDs**. A) SK19 cells treated for 3 h with drugs as described in Methods. Cells were fixed and subjected to immunofluorescence using a monoclonal anti-20S proteasome subunit α2 primary antibody, followed by incubation with the Alexa Fluor^® ^647 goat anti-mouse secondary antibody. Right panels represent colocalization of GFP-ERα and 20S proteasome (*insets*): untreated cells (4A,*c*) and 10nM E2 treated cells (4A,*f*),. Images are representative of three independent experiments. Bar, 5 μm. B) 80 nm thin sections of MCF-7 cells were incubated with anti-ERα and anti-20S proteasome subunit α2 antibodies. Secondary antibodies coupled with 10 nm and 6 nm gold particles were used against anti-ERα (white arrowhead) and anti-20S proteasome (black arrowhead), respectively. Grids were then processed for electron microscopy. Colocalization of both proteins is indicated by circles. Bar, 500 nm. The area of selected electron micrograph shows colocalization clusters where at least 3 gold particles for each protein are present (inset). Bar, 100 nm. C) Western blot showing SERD-mediated degradation of ERα through the nuclear proteasome. SK19 cells were pre-treated or not with inhibitor of nuclear export (10 nM LMB) or proteasome inhibitor (100 μM ALLN) for 30 min and then treated with vehicle (EtOH as reference), 10 nM E2, 1 μM ICI, or 1 μM RU58 for 3 h. ERα and GAPDH (internal control) detection by Western blotting shows proteasome-dependent degradation of ERα upon E2 and SERDs stimulation. D) SK19 cells treated for 3 h with SERDs were pre-treated or not with proteasome inhibitor ALLN (details see in Methods). Right panels represent colocalization of GFP-EPα and 20S proteasome subunit α2 (*insets*): SERDs pre-treated cells without (4D*c *and *i*) or with (4D*f *and *l*) 100 μM ALLN. Images are representative of three independent experiments. Bar, 5 μm.

The 26S proteasome is a large protein complex (1500-2000 kDa) present in the cytoplasm and nucleus of eukaryotic cells. The catalytic core of this multi-subunit complex, described as the 20S proteasome, contains α and β subunits [33]. We visualized GFP-ERα and the 20S proteasome subunit α2 in SK19 cells. SK19 cells grown on glass coverslips and treated as described were fixed, permeabilized and subjected to indirect immunofluorescence using a monoclonal anti-20S proteasome subunit α2 primary antibody. Images acquired on an Olympus inverted wide-field microscope in 3 D and subjected to deconvolution revealed punctuate nuclear staining of proteasome subunits throughout the nucleus (Figure 4A and 4D, center panels). We did not observe any cytoplasmic staining of this proteasome subunit under our culture conditions. In the presence of E2, GFP-ERα accumulated at numerous nuclear sites that colocalized at least partially with proteasome foci (Figure 4A panel *f*).

Next we used a double-immuno-nanogold labelling approach in MCF-7 cells to characterize the extent of ER/α2 colocalization. Upon exposure to E2, at least four nuclear clusters per nuclear sections were detected. In the majority of clusters more than 3 gold particles for each protein were present (as indicated by circles in Figure 4B). Endogenous ERα (represented by 10 nm gold particles) colocalized with the 20S proteasome subunit α2 (represented by 6 nm gold particles) in nuclear microdomains of about 100 nm in diameter (Figure 4B, inset).

We then determined the effect of LMB (Leptomycin B), an inhibitor of the nuclear export receptor CRM1, and of ALLN (acetyl-leucyl-leucyl-norleucinal), an inhibitor of the proteasome, on SERD-dependent degradation of ERα in SK19 cells. SK19 cells were pretreated with 10 nM LMB or 100 μM ALLN for 30 min. Figure 4C shows that LMB did not block E2, ICI or RU58 induced ERα degradation suggesting that SERD-bound ERα is degraded in the nucleus. In the presence of E2, but not ICI or RU58, degradation was slightly less pronounced in cells pretreated with LMB suggesting that a fraction of E2 bound ERα is also degraded by the cytoplasmic proteasome. Furthermore, ALLN inhibited E2, ICI and RU58 induced degradation of ERα confirming that SERD-ERα complexes were degraded by the nuclear proteasome (Figure 4C, *lanes 6, 9 and 12*). Note that at the protein level, GFP-ERα is degraded to a lesser extent than endogenous ERα which is likely to be a consequence of reduced transcription of ESR1 in the presence of E2 and SERDs. GFP-ERα transcription is under the control of a CMV promoter which insensitive to antiestrogens.

Finally, we investigated the distribution of GFP-ERα and the 20S proteasome subunit α2 in SK19 cells treated with ICI or RU58 (Figure 4D). GFP-ERα foci also significantly overlapped with accumulation sites of the 20S proteasome subunit α2 throughout the nucleus (Figure 4D, insets of panels *c *and *i*). On average we observed larger and more frequent GFP-ERα-proteasome complexes in the presence of SERDs than in the presence of E2 consistent with the fact that ERα is readily degraded when ERα is bound to SERDs. As a consequence of the ALLN treatment, contacts between GFP-ERα and proteasome foci were largely abolished (Figure 4D panels *f *and *l*).

Interestingly, in a few cells treated with either E2 or SERDs we observed a single very large site of accumulation of the 20S proteasome α2 subunit (data not shown). These sites, also called *clastosomes*, were reported to colocalize with the c-jun and c-fos proteins [34], very unstable proteins with half lives of less than 90 min. In our cells, clastosomes did not colocalize with GFP-ERα foci which may indicate that E2 bound ERα is more stable than c-jun and/or c-fos proteins.

## Discussion

The available quantity of ERα is a limiting factor in the response to ligands, estrogen and antiestrogens. Thus, determination of ERα cell content in patients is not only the first parameter for tumour classification, but also a powerful tool to predict response to hormone-therapies. ERα protein levels vary under physiological states, during tumor progression, and beyond therapy [10,35,36]. ERα protein levels are tightly regulated by the ubiquitin-proteasome pathway and loss of this control is associated with hormone insensitivity in breast cancer [37].

Most members of the nuclear receptor superfamily form focal accumulations within the nucleus in response to hormone [38]. Receptors undergo constant exchange between target sequences, multi-protein complexes including a variety of transcription factors, as well as subnuclear structures that are as yet poorly defined. The estrogen receptor alpha is found almost exclusively in the nucleus, both in hormone stimulated and untreated cells which makes it an exception among nuclear receptors which generally translocate from the cytoplasm into the nucleus upon hormone stimulation. Hager and colleagues [38] proposed that distribution of the ERα is dependent not only on localization signals, but also on the nature and composition of the associated macromolecular complexes. Formation of these complexes depends on the nature of the ligand bound to ERα. Thus, as demonstrated here, ligands directly affect the nuclear fate of the receptor.

We created a MCF-7 cell line stably expressing GFP-tagged human ERα to levels equivalent to endogenous ERα, to determine the localization of ligand-bound GFP-ERα in mammary tumor cells. We demonstrate that few hours after treatment cellular localization of the ERα correlates with the nature of the ligand independently of its impact on transcription.

In the presence of E2 and SERMs which induce binding of ERα to target sequences and subsequent formation of macromolecular complexes, the small cytoplasmic fraction of E2 bound ERα rapidly translocated into the nucleus suggesting that DNA binding attracts cytoplasmic ERα. In contrast, SERD bound cytoplasmic ERα was retained in the cytoplasm. SERDs induce a conformational change of ERα independently of its localization (cytoplasmic or nuclear) which leads to its rapid degradation. Our data also corroborate recent observations by Long and co-workers [39,40] that ICI induces specific nuclear matrix interaction of protein-ERα complexes with cytokeratins 8 and 18 which mediate immobilization and turnover of ERα. A non direct role of ERα in the cytoplasm has been proposed to play a role in acquired resistance to antiestrogens, in particular OHT [41]. Indeed, in OHT resistant cells, the ERα accumulated in the cytoplasm, suggesting that SERM stimulated ERα relocalization into the nucleus may be necessary for anti-hormone effectiveness (through the modulation of macromolecular complexes bound to the ERα). An attractive possibility would thus reside in not only blocking indirect ERα functions which rely on MEK/ERK and PI3K pathways in SERM resistant tumors, but to increase ERα translocation into the nucleus.

The crystal structure of ERα bound to different ligands has revealed a spectrum of conformational states [9,42-44] that involve the repositioning of helix H12 of the receptor's ligand binding domain and formation the receptor's cofactor associating surfaces. It was proposed that the ligand binding cavity has a remarkable plasticity with a preferential binding mode for distal hydroxyl groups [43] showing similar orientations for distal side chains in α or β positions of different ligands [43]. RU39 and RU58 are derivatives of 17β-estradiol but with different side chains. The shorter dimethyl-amino-ethoxy-phenyl side chain is similar to the one in 4-hydroxytamoxifen and likely to be easily accommodated by the cavity (Mazaheri et al in preparation). In contrast, RU58 has a bulky hydrophobic side chain similar to the one in fulvestrant (ICI) which hampers the folding of helix 12. Thus the molecular structure of ERα ligands alone indicates the potential for SERM or SERD -like activities of the compound (Mazaheri et al. in preparation).

Interestingly, E2 induced focal accumulations of ERα scattered throughout the nucleus in the presence of E2 and of SERDs (Figure 3A and 4A, D). In agreement with this observation, numerous ERα-rich domains of about 100 nm are detectable following E2 stimulation (data not shown). It is well established that upon E2 addition, ERα binds to promoter of ERα-target genes [45]. Stimulated genes are found at numerous sites in the nucleus similarly to ERα protein [46]. Thus, we propose that the observed ERα rich nuclear clusters correspond to association of the receptor with chromatin structures of ERα-responsive genes and the proteasome (Figure 3Ae and 4B) to ensure its own turnover while target genes are being transcribed. Similarly, SERD-bound ERα also concentrated into nuclear foci (Figure 3Ah, k and 3B) which frequently colocalize with the proteasome independently of DNA binding. This may explain why ligand bound ERα is less dynamic, and appears more strongly associated with nuclear matrix like structures [27].

Thus we propose a simple explanation reconciling all previous observations of ERα dynamics: ligands that allow ERα to bind its target sequence and to recruit macromolecular complexes induce ERα nuclear degradation or accumulation (E2 or SERMs); ligands that bind to ERα but do not lead to DNA binding due to conformational changes of the receptor do not induce relocalization of the receptor, but accelerate its degradation (SERDs); finally, ligands that induce association of ERα with the proteasome (E2 and SERDs) lead to focal accumulations and immobilize the ERα. It is the association with the proteasome [47] and not active degradation by the proteasome that leads to ERα sequestration.

In the last 30 years clinical use of tamoxifen significantly improved survival rates of patients with hormone-dependent breast cancer types. However, resistance to this therapy arises frequently and numerous side effects exist. Since it is well established that total ERα content correlates with tumor growth in response to different ligands, it is crucial to characterize the exact mechanisms involved in anti-estrogen action and the impact of their structure on ERα conformation, co-factor recruitment and cellular compartmentalization. Knowledge of these parameters may allow to develop new compounds useful for patients resistant to existing therapies but may also benefit early diagnostics and treatment design.

## Conclusions

In conclusion the results of this study indicate the impact of the estradiol and several SERM and SERD compounds, in particular RU39,411 and RU58,668, on nucleocytoplasmic shuttling and protein turnover of estrogen receptor alpha (ERα) in human breast cancer cell lines. We found that ligands directly affect the nuclear fate and protein turnover of the receptor independently of their impact on transcription.

## Methods

### Reagents

17β-estradiol (E2), 4-hydroxytamoxifen (OHT) and Leptomycine B (LMB) were purchased from Sigma-Aldrich (St. Louis, MO). ICI 182,780 (ICI) was purchased from Zeneca Pharmaceuticals. RU39,411 (RU39) and RU58,668 (RU58) were kindly provided by Dr. J.M. Renoir (Paris, France). Stock solutions of E2, OHT, ICI, RU39 and RU58 were prepared in ethanol. Stock solution of LMB was prepared in methanol. The solution of proteasome inhibitor acetyl-leucyl-leucyl-norleucinal (ALLN) was purchased from Calbiochem.

Rabbit polyclonal anti-ERα (HC-20), rabbit polyclonal anti-lamin A (H-102), rabbit polyclonal anti-cytokeratine 18 (H-80) were purchased from Santa Cruz Biotechnology, Inc. Mouse monoclonal anti-GAPDH (MAB374) was purchased from Chemicon International, mouse monoclonal anti-GFP from Roche, mouse monoclonal anti-α-tubulin (clone DM1A) from Sigma-Aldrich. Mouse monoclonal anti-20S proteasome subunit α2 (clone MCP21) was gift from Dr. M.P. Bousquet (IPBS, Toulouse, France).

All cell culture products were obtained from Invitrogen.

### Cell culture and generation of stable GFP-ERα cell line

Human breast cancer cell lines were maintained in Dulbecco's modified Eagle's medium F-12 (DMEM F-12) with Glutamax containing 50 μg/ml gentamicin, 1 mM sodium pyruvate and 10% heat-inactivated fetal calf serum. All cells were grown at 37°C in a humidified atmosphere containing 5% CO_2_. The stably transfected GFP-ERα reporter SK19 cell line was generated from ERα-positive breast cancer MCF-7 cells (ATCC). 2nd passage cells were transfected with a GFP-ERα expression vector (pEGFP-C2-hERα) using FuGENE^® ^HB Transfection Reagent (Roche Applied Science) and G418 resistant clones were selected at the concentration 1 mg/ml. GFP-ERα expressing clones were isolated, ERα protein expression in response to estradiol and to anti-estrogens was quantified using fluorescence microscopy and western blot. Expression of ERα-regulated genes was tested by qRT-PCR and compared to gene-expression regulation in MCF-7 cells. The clone SK19 in which GFP-ERα behavior was comparable to endogenous ERα was selected for further investigation.

To study the effects of estrogens and antiestrogens, cells were grown for 3 days in medium containing phenol red-free DMEM F-12 supplemented with 5% charcoal-stripped fetal calf serum, without gentamicin and sodium pyruvate. Cells were subsequently treated or not with 10 nM E2, 1 μM ICI, 1 μM OHT, 1 μM RU39, 1 μM RU58 for the indicated times. To study ERα degradation by the proteasome, cells were pre-treated 30 min with 100 μM ALLN, a proteasome inhibitor, or 10 nM LMB, a nuclear export inhibitor.

### Cell extracts and Western blots

MCF-7 cells grown in 6-well plates were treated as indicated, washed with ice-cold PBS and collected by centrifugation. Total cell lysates were prepared by resuspension of cells in 100 μl lysis buffer (50 mM Tris pH = 6.8, 2% SDS, 5% glycerol, 2 mM EDTA, 1.25% β-mercaptoethanol, 0.004% Bromophenol blue). The samples were boiled for 20 min at 95°C and cleared by centrifugation at 12 000 × *g *for 10 min. Protein concentration was determined by an Amido Schwartz assay when the samples contained SDS. Samples were subjected to SDS-PAGE and proteins transferred onto nitrocellulose membranes. Western blot analysis was performed as previously described [48] using ERα and GAPDH antibodies and quantified using the TINA PC-Base Software from FUJI.

### qRT-PCR experiments

Total RNAs were extracted using TRIzol reagent (Invitrogen) following the manufacturer's protocol. 1-5 μg of total RNA was reverse transcribed in a final volume of 20 μl using SuperScript™III Reverse Transcriptase (Invitrogen). cDNA was stored at -80°C. All target transcripts were detected using quantitative RT-PCR (SYBRGreen SuperMix, Invitrogen) assays on a Mastercycler Realplex device (Eppendorf) using *TBP *or *RPLP0 *genes as endogenous control for normalization of the data. The following primer pairs were used for amplification:

*TBP*: 5'-CGGCTGTTTAACTTCGCTTTC-3'

5'-CCAGCACACTCTTCTCAGCA-3'

*ESR1*: 5'-TGGAGATCTTCGACATGCTG-3'

5'-TCCAGAGACTTCAGGGTGCT-3'

*GREB1*: 5'-GTGGTAGCCGAGTGGACAAT-3'

5'-AAACCCGTCTGTGGTACAGC-3'

*RPLP0*: (Fwd) 5'-TGGCAGCATCTACAACCCTGAA-3'

(Rev) 5'-ACACTGGCAACATTGCGGACA-3'

*TFF1: *(Fwd) 5'-CCCCTGGTGCTTCTATCCTAAT-3'

(Rev) 5'-CAGATCCCTGCAGAAGTGTCTA-3'

*PGR: *(Fwd) 5'-CTTAATCAACTAGGCGAGAG-3'

(Rev) 5'-AAGCTCATCCAAGAATACTG-3'

The results were analyzed using Mastercycler Realplex and qBASE software.

### Cell fractionation

Three hours after incubation with ERα ligands, SK19 cells were washed with ice-cold PBS, scraped and centrifuged at 1,500 rpm for 5 min at 4°C. The pellets were resuspended in 150 μl digitonin lysis buffer containing 1% digitonin and 1 mM EDTA in PBS, immediately centrifuged at 13,000 rpm for 20 min at 4°C, to obtain the cytosolic fraction (C). The pellets were resuspended in 150 μl HEPES lysis buffer containing 1% Triton X-100, 10% glycerol, 10 μg/ml leupeptin, 5 μg/ml aprotinin, 1 mM PMSF, 1 mM Na_3_VO_4 _and 50 mM NaF in HEPES buffer (25 mM HEPES, 0.3 M NaCl, 1.5 mM MgCl_2_, 20 mM β-glycerol-phosphate, 2 mM EDTA, 2 mM EGTA and 1 mM DTT), kept 15 min on ice and centrifuged at 13,000 rpm for 15 min at 4°C, to obtain the soluble nuclear fraction (SN). The pellets from the previous step were resuspended in 100 μl of a third buffer containing 95% Laemmli buffer and 5% β-mercaptoethanol and incubated 5 min on ice and boiled for 20 min at 95°C to obtain the insoluble nuclear fraction (IN). The different fractions were stored at -80°C until use. Protein concentrations were determined using the Bio-Rad Protein Assay (Bio-Rad).

### Immunofluorescence and Fluorescence microscopy

For indirect immunofluorescence experiments, SK19 cells were grown for 3 days on coverslips in DMEM without phenol red, containing 5% charcoal-stripped fetal serum. After 3 days, cells were treated for 1 h with the following ligands: 10 nM E2, 1 μM ICI, 1 μM OHT, 1 μM RU39, 1 μM RU58. Cells were then washed twice with PBS, fixed in 4% paraformaldehyde/PBS for 10 min at room temperature, subsequently permeabilized with 0.5% Triton X-100 in PBS for 15 min at room temperature, counterstained with DAPI (4',6-diamidino-2-phenylindole) and mounted on microscopy slides.

To study co-localization of ERα and proteasome by immunofluorescence, SK19 cells were grown for 3 days on coverslips in DMEM without phenol red, containing 5% charcoal-stripped fetal serum and next treated for 3 h with drugs as indicated above. To block proteasome-mediated ERα degradation, the cells were incubated 30 min with 100 μM ALLN prior to treatment with ICI or RU58. Before immunostaining, cells were fixed in 4% paraformaldehyde/PBS for 30 minutes at room temperature, washed three times in PBS, quenched in 75 mM NH_4_Cl containing 20 mM glycine and permeabilized with 0.5% Triton X-100 in PBS for 30 minutes. Next, cells were washed with PBS, blocked for 1 h at room temperature in 5% dry milk in TBS-T (20 mM TRIS-HCl, 150 mM NaCl, 0.1% Tween 20, pH = 7.4) and incubated overnight at 4°C with anti-20S proteasome antibody at a final concentration 2 μg/ml in 5% dry milk in TBS-T followed, after washing, by incubation with the Alexa Fluor^® ^647 goat anti-mouse secondary antibody (1:1000, Invitrogen, Molecular Probes) for 90 min in the dark at room temperature. Finally, cells were washed with TBS-T, counterstained with DAPI and mounted on microscopy slides.

Cells were examined by fluorescence microscopy using an Olympus IX-81 microscope, equipped with a CoolSNAP HQ camera (Roper Scientific) and imaged through an Olympus oil-immersion objective 100x PLANAPO NA1.4. Images were recorded and deconvolved using Metamorph software (Universal Imaging). All images were processed for presentation using Adobe Photoshop 9.0.2.

### Electron microscopy

MCF-7 cells were grown and treated as described above. For immune-electron microscopy cells were fixed with 4% paraformaldehyde in Na cacodylate buffer (pH 7.4), dehydrated in a graded series of ethanol and embedded in acrylic resin (LRWhite). 80 nm ultrathin sections were mounted on Nickel grids, incubated with 2% BSA/PBS and incubated overnight at 4°C with a mixture of primary antibodies (anti -20S proteasome antibody at final concentration 2 μg/ml and anti-ERα antibody (dilution 1/500)) in 2% BSA/PBS, washed 5 times for 5 mins in 1% BSA/PBS and then labeled for 1 h with 6 nm goat anti-mouse and 10 nm goat anti-rabbit gold conjugated particles in 1% BSA/PBS. Grids were finally washed 4 times for 5 mins in 1% BSA/PBS, incubated for 15 mins in 1% glutaraldehyde/PBS, washed 2 times for 5 mins in PBS, 3 times in distilled water and dried at room temperature. The samples were visualized using 120 kV Jeol electron microscope at 80 kV and images were captured using a digital camera AMT.

## Competing interests

The authors declare that they have no competing interests.

## Authors' contributions

SK created SK19 cell line, designed experiments, performed and analyzed immuno-electron and fluorescence microscopy data; MM designed experiments, performed and analyzed western blot and qRT-PCR experiments; SCS did the cell fractionation experiments; KB designed experiments, participated in data analysis and provided lab support; SK, MM and KB wrote the paper. All authors read and approved the final manuscript.
